# Management Challenge: An Atypical Variant of Takotsubo Presenting With Multiple Complications

**DOI:** 10.7759/cureus.26836

**Published:** 2022-07-14

**Authors:** Kristina Menchaca, Catherine A Ostos Perez, Nemanja Draguljevic, Shaun Isaac

**Affiliations:** 1 Internal Medicine, University of Miami/John F. Kennedy (JFK) Medical Center, Atlantis, USA; 2 Internal Medicine, University of Belgrade, Belgrade, SRB; 3 Internal Medicine, John F. Kennedy (JFK) Medical Center, Atlantis, USA

**Keywords:** takotsubo and sinus node dysfunction, cardiac arrhythmia and takotsubo cardiomyopathy, global hypokinesis, pacemaker, sinus node dysfunction, acute mitral valve regurgitation, cardiogenic shock, atypical takotsubo, takotsubo cardiomyopathy (ttc)

## Abstract

An 84-year-old woman with depression, who witnessed the suicide of a close friend, presented with symptoms of chest pain, palpitations, and cold and clammy extremities. An electrocardiogram showed alternating tachycardia and bradycardia. Urgent transthoracic echocardiogram demonstrated left greater than right ventricular dysfunction, moderate mitral regurgitation, global hypokinesis, and an estimated ejection fraction of 20%. Cardiac catheterization demonstrated non-obstructive coronary artery disease and decreased cardiac output. Findings were consistent with Takotsubo cardiomyopathy complicated with cardiogenic shock, acute mitral regurgitation, and sinus node dysfunction. Management of this patient required the use of a mechanical device intra-aortic balloon pump, and pacemaker insertion for persistent symptomatic arrhythmia. This case highlights the challenging management of potentially fatal acute complications of Takotsubo cardiomyopathy and inadequate data on how to approach them.

## Introduction

Takotsubo cardiomyopathy (TCM), also known as stress-induced cardiomyopathy, represents transient systolic and diastolic left ventricular dysfunction [1]. Clinical manifestation is similar to acute coronary syndrome; however, wall motion abnormality extends beyond coronary artery supply regions [1,2]. The typical form of TCM manifests with hypokinesis of the mid and apical segments, and with hyperkinesis of the basal segments, leading to an “octopus trap” appearance on the left ventriculogram. Takotsubo can also present with other forms called "atypical variants" such as the inverted Takotsubo or basal variant, with circumferential basal hypokinesia and apical hypercontractility [1,2]. In this case, TCM presented with global hypokinesis.

TCM has usually been associated with good outcomes and conservative management is effective [1,2]. However, the development of complications carries a higher risk of morbidity and mortality [1-3]. There are no standardized guidelines in regards to the diagnostic and management approach. Here, we report a case of an atypical variant of Takotsubo with a global hypokinesis pattern complicated by cardiogenic shock, mitral valve regurgitation, and sinus node dysfunction (SND).

## Case presentation

An 84-year-old Caucasian woman with a medical history of depression presented to the emergency department with palpitations, mid-sternal chest pain, and dyspnea for two days. One week before the development of the symptoms, the patient witnessed the suicide of a close friend. She denied previous cardiac history. Vitals included a blood pressure of 97/51, heart rate of 136 beats/minute, respiratory rate of 18 breaths/minute, oxygen saturation greater than 95% on room air, and the patient was afebrile. Estimated jugular venous pressure was 9 cm above the sternal angle. A physical exam revealed bilateral lung crackles and a 3/6 systolic murmur in the 4th left intercostal space. The lower extremities were cold and clammy.

**Figure 1:**
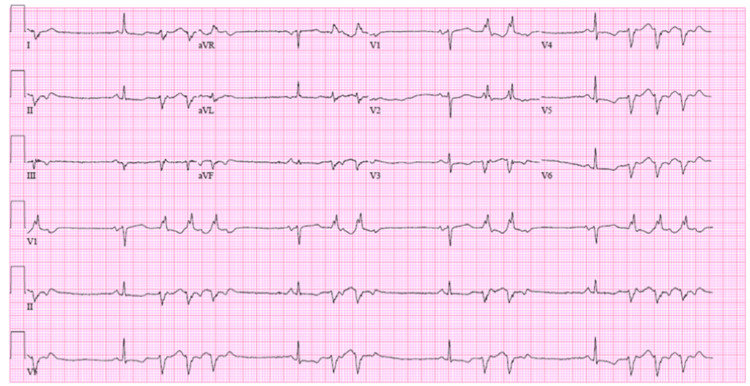
Electrocardiogram findings showing sinus bradycardia with premature ventricular complexes.

**Figure 2:**
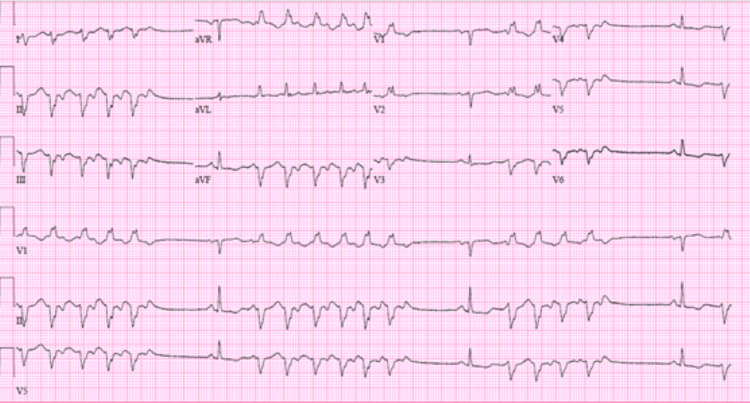
Sinus tachycardia with frequent and consecutive premature ventricular contractions.

An initial electrocardiogram (EKG) showed sinus bradycardia and frequent premature ventricular contractions (PVCs) (Figure 1). The subsequent EKG, which was performed after 10 minutes, demonstrated sinus tachycardia and PVCs (Figure 2).

**Figure 3:**
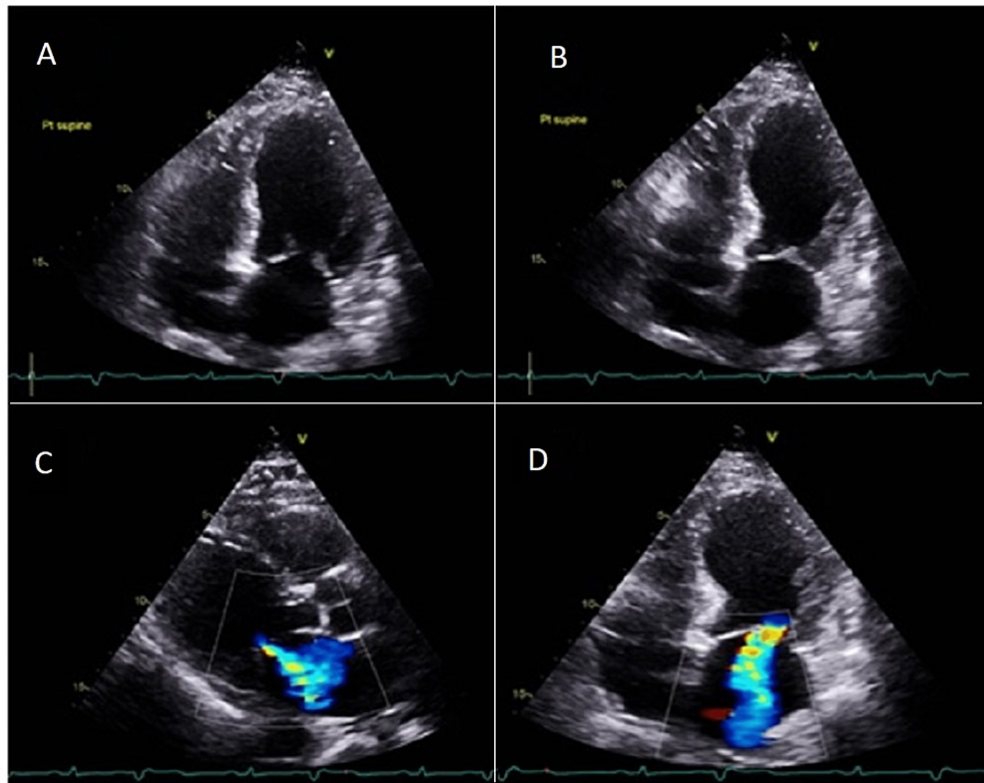
Transthoracic echocardiogram apical four-chamber view in diastolic (A) and systolic (B) period and parasternal (C) and four-chamber apical view (D) demonstrating moderate mitral regurgitation and global hypokinesis.

Initial troponin I was 0.217 ng/ml, and increased to 3.140 ng/ml (positive > 0.1 ng/ml). Urgent transthoracic echocardiogram (TTE) demonstrated an ejection fraction (EF) of 20% with left greater than right ventricular dysfunction, moderate mitral regurgitation, and global hypokinesia (Figure 3).

**Figure 4:**
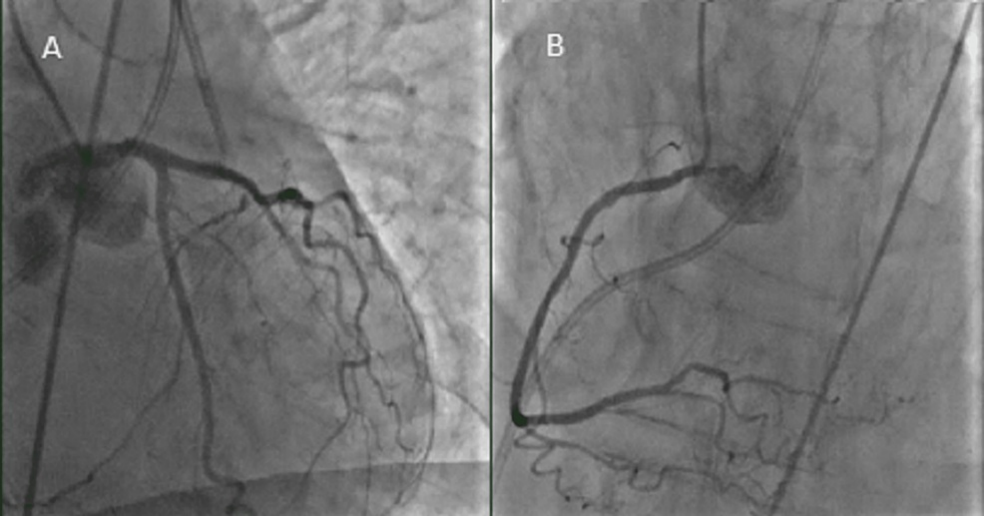
Coronary angiography revealing normal coronary arteries without significant stenosis. (A) Left coronary artery. (B) Right coronary artery.

**Figure 5:**
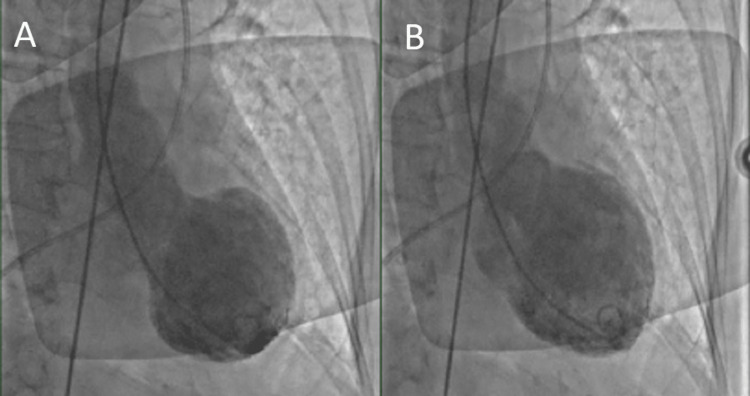
Left ventriculogram: (A) systole and (B) diastole.

This prompted cardiac catheterization, which revealed nonobstructive coronary artery disease (Figure 4). The left ventriculogram demonstrated severely depressed global systolic dysfunction, and moderate mitral regurgitation suggesting TCM (Figure 5). Hemodynamic measurements included pulmonary capillary wedge pressure of 27 mmHg, cardiac output of 2.3 L/min, cardiac index of 1.5 L/min/m^2^, and pulmonary artery oxygen saturation of 39.9%.

At this stage, the patient was diagnosed with Takotsubo manifested with global hypokinesis and complicated with cardiogenic shock and new-onset mitral regurgitation. Left ventricular outlet obstruction was excluded and we inserted an intra-aortic balloon pump (IABP) for hemodynamic support. On the fourth hospital day, IABP was removed due to improvement of cardiac index and output. On the sixth hospital day, the patient's shock was resolved, and the use of vasopressors was discontinued. Cardiac magnetic resonance showed severe global hypokinesis with a reported EF of 27%. Late gadolinium enhancement was absent.

Despite conservative management with amiodarone and metoprolol, the patient continued to experience symptomatic alternating bradycardia and non-sustained ventricular tachycardia for several days; therefore, anti-arrhythmic medical therapy was not effective. An electrophysiologist was consulted to evaluate the patient’s arrhythmia and a pacemaker was placed. Heart failure-guided pharmacological treatment was started and the patient was discharged from the hospital in stable condition. Repeated TTE several weeks later revealed normal left ventricular function with EF of 55% and resolved mitral valve regurgitation. She remained asymptomatic and without heart failure-related symptoms as well as palpitations.

## Discussion

Traditionally, a depressed left ventricular function associated with TCM resolves over several weeks and has a good long-term prognosis [1,2]. However, the potential development of fatal complications, such as acute heart failure, cardiogenic shock, arrhythmias, and left ventricular outflow tract obstruction (LVOTO), are major determinants of the outcome [1-4].

Therefore, the management of several concomitant complications such as cardiogenic shock, mitral regurgitation, and SND can be challenging. Furthermore, this was observed in the context of a rarely reported echocardiogram variant of Takotsubo such as global hypokinesis.

Left ventricular failure causing cardiogenic shock may be worsened by mitral regurgitation, LVOTO, and right ventricular involvement. Therefore, early recognition of the underlying mechanism of cardiogenic shock and associated LVOTO with echocardiography plays a central role in guiding decisions for appropriate management [1-5]. The choice of a mechanical device for hemodynamic support in cardiogenic shock relies on excluding LVOTO because the use of certain mechanical devices such as Impella (Abiomed, Danvers, MA) may worsen shock [1-6]. Associated acute mitral regurgitation is detected in 14% of the cases [1-6]. Apical tethering, observed in our case, can cause reversible mitral regurgitation in patients with Takotsubo.

Patients with TCM may also develop life-threatening conduction abnormalities, which should be closely monitored in the acute phase [7,8]. Some of the reported arrhythmias are asystole, pulseless electrical activity, complete sinoatrial and atrioventricular block, ventricular tachycardia, and ventricular fibrillation [7,8]. SND is reported in the context of TCM, however, with an estimated incidence of 1.3% [7-9]. SND may be induced by Takotsubo, or SND by itself can trigger TCM, which is explained by adrenergic compensatory activation following bradycardia events [7-9].

Our patient developed alternating episodes of non-sustained ventricular tachycardia and bradycardia, which subsequently became persistent and symptomatic despite medical management with amiodarone and beta-blocker. It is unclear if rhythm disorder was caused by underlying subclinical SND or if Takotsubo was a triggering event. However, the patient had identified strong emotional triggers preceding the event, but the coincidence is also that post-menopausal women belong to the age-related risk category for SND.

Managing arrhythmias associated with TCM with a pacemaker or implantable cardioverter-defibrillator (ICD) is controversial due to the reversibility of the disease [7-9]. We decided to place a pacemaker due to the persistence of symptoms related to arrhythmia and failure of conservative management. Although our patient underwent pacemaker insertion, more data are needed to identify patients who might benefit from ICD or pacemaker implantation.

## Conclusions

This case illustrates the importance of early recognition and management approach to acute complications of TCM to prevent potentially fatal outcomes. Their occurrence is a challenge and there are no established guidelines on how to approach them. Arrhythmias in Takotsubo are recognized as one of the determining factors of morbidity and mortality, and close monitoring is required. Furthermore, this case highlights the importance of future studies to address drug therapy or the inclusion of invasive pacemakers and ICDs in the management of arrhythmias in Takotsubo.
